# A Self-Adaptive Progressive Support Selection Scheme for Collaborative Wideband Spectrum Sensing

**DOI:** 10.3390/s18093011

**Published:** 2018-09-08

**Authors:** Zhuhua Hu, Yong Bai, Mengxing Huang, Mingshan Xie, Yaochi Zhao

**Affiliations:** 1 College of Information Science & Technology, Hainan University, Haikou 570208, China; eagler_hu@hainu.edu.cn (Z.H.); huangmx09@163.com (M.H.); hnmingshanxie@163.com (M.X.); yaochizi@163.com (Y.Z.); 2 State Key Laboratory of Marine Resource Utilization in South China Sea, Hainan University, Haikou 570208, China

**Keywords:** cognitive radio network, singular value decomposition, cooperative wideband spectrum sensing, transmission loss, modulated wideband converter, progressive support selection

## Abstract

The sampling rate of wideband spectrum sensing for sparse signals can be reduced by sub-Nyquist sampling with a Modulated Wideband Converter (MWC). In collaborative spectrum sensing, the fusion center recovers the spectral support from observation and measurement matrices reported by a network of CRs, to improve the precision of spectrum sensing. However, the MWC has a very high hardware complexity due to its parallel structure; it sets a fixed threshold for a decision without considering the impact of noise intensity, and needs a priori information of signal sparsity order for signal support recovery. To address these shortcomings, we propose a progressive support selection based self-adaptive distributed MWC sensing scheme (PSS-SaDMWC). In the proposed scheme, the parallel hardware sensing channels are scattered on secondary users (SUs), and the PSS-SaDMWC scheme takes sparsity order estimation, noise intensity, and transmission loss into account in the fusion center. More importantly, the proposed scheme uses a support selection strategy based on a progressive operation to reduce missed detection probability under low SNR levels. Numerical simulations demonstrate that, compared with the traditional support selection schemes, our proposed scheme can achieve a higher support recovery success rate, lower sampling rate, and stronger time-varying support recovery ability without increasing hardware complexity.

## 1. Introduction

Spectrum resources have become increasingly scarce with emerging wireless services. Nevertheless, assigned radio spectrums to authorized users are mostly underutilized. As a solution to this problem, cognitive radio (CR) technology can reuse spectrum resources by utilizing spectrum sensing to intelligently recognize idle frequency bands [1]. Traditional spectrum sensing methods, such as energy detection [2], cyclostationary feature detection [3], and matched filter detection [4], mainly exploit spectral opportunities over a narrow frequency range. The research of wideband compressed spectrum sensing (WCSS) is motivated by the desire to support wireless multimedia communications in CR networks [5,6]. In WCSS, compressed sensing (CS) theory [7,8] can be applied to reduce the sampling rate and the hardware complexity of the CR transceivers, such as by using a wideband antenna, wideband filter, and high speed analogue-to-digital converter (ADC).

In the early years, CS research has mainly concentrated on the sensing of discrete finite-length signals [9]. Recently, CS was applied in the analog domain to sample the wideband analog signals at rates far lower than the Nyquist sampling rate [10]. There have been several hardware architectures proposed for continuous-time signals, such as the Analog-to-Information Conversion (AIC) system [11,12], Multi-Coset (MC) system [13,14,15], and Modulated Wideband Converter (MWC) system [16,17,18]. The AIC system is designed for narrowband multi-tone signals, while the MC and MWC systems have multi-channel structures and can reduce the sampling rate significantly for wideband multi-band signals. Unfortunately, the MC system needs high-rate sampling in the analog front-end, and the time delay of each channel must be accurate. The MWC is an attractive wideband spectrum sensing technique for multi-band signals, which requires m parallel sampling channels, with each channel comprised of a modulator with a mixing function $p_{i}(t)$, a low-pass filter (LPF) and an ADC. However, the MWC has high hardware complexity owing to a large number of parallel channels. If m is to be reduced enough without increasing the sampling rate at each channel, the sensing performance would be degraded sharply. Therefore, most of the recent papers about the MWC focus on how to improve the reconstruction accuracy of the spectral support [19,20,21,22] and how to design novel hardware architecture with low complexity [23,24,25,26]. 

The signals sensed by CR may suffer from fading during transmission, and the performance of a single CR can be affected. To improve the precision of spectrum sensing, collaborative spectrum sensing can be conducted by multiple CRs, where different CRs share their sensing results and cooperatively decide on the spectrum occupancy [27]. In centralized collaborative spectrum sensing, multiple CRs report their measurements to a fusion center, which makes a joint decision on the spectral support [28]. In [29], the MWC is used in the distributed cooperative spectrum sensing (DCSS), in which each CR node uses the MWC system to sample a wideband sparse signal, where a secondary user (SU) can be considered as a CR node. However, this strategy can lead to unacceptable hardware complexity and cost. Xu et al. proposed a distributed MWC (DMWC) scheme, which regards one CR node as one sampling channel [30]. The DMWC scheme can reduce the complexity and the cost of the single sensing node. However, the DMWC needs a large number of cooperative SUs to maintain a high success recovery probability, and has a poor sensing performance under low SNR. In addition, the DMWC needs to know the prior information of sparsity order. Unfortunately, in practical CR applications, the real sparsity order is time varying in nature. Thus, due to the distinct radio wave propagation environment, the traditional WCSS technologies cannot be applied directly to sense the wideband spectrum between SUs. To address above problems, in this paper a novel progressive support selection based self-adaptively distributed MWC sensing scheme (PSS-SaDMWC) is proposed. Our main contributions are summarized as follows:

(1) To reduce the complexity and the cost of cooperative wideband spectrum sensing, inspired by [30] in which the parallel hardware sensing channels are scattered on various SUs. Namely, the ith secondary user is treated as the ith compressed sampling channel. To mitigate multipath fading in the CR environment, we build the transmission loss model. In the fusion center (FC), the under-sampling data from SUs is multiplied by the transmission loss gain. Moreover, the influence of transmission loss on the support recovery performance is also discussed.
(2) To self-adaptively achieve a high successful reconstruction probability of spectral support in a practical CR environment. Firstly, according to the theory of singular value decomposition [31], the internal relationship between noise singular values and useful signal singular values in a noisy signal is obtained. Then, because the tail singular values are mainly determined by noise, and the noise singular value has a linear characteristic, the proposed scheme uses the linear relationship to estimate the noise intensity by adding a known additive white Gaussian noise (AWGN) signal in the FC. Thirdly, with the contribution of the estimated noise singular values to the singular values of the noised-signal, we use a gradient and difference operation to estimate the sparsity order.
(3) Given a false alarm probability ($P_{f}$) and transmission interference, the scheme uses the progressive support selection strategy to significantly decrease the probability of missing detection under low SNR levels.

The rest of this paper is organized as follows: Section 2 describes the sampling principles of the MWC and cooperative sensing system model, and gives the problem statement. Section 3 presents the proposed PSS-SaDMWC scheme, including the analysis of transmission loss, the estimation of noise intensity and sparsity order, and the discussion of the progressive support selection strategy. In Section 4, we conduct a numerical evaluation of the proposed scheme and discuss the simulation results. Finally, Section 5 concludes the paper. The main notations used are listed in Table 1.

## 2. System Model and Problem Statement

### 2.1. Basic Principle of the MWC

**Figure 1:**
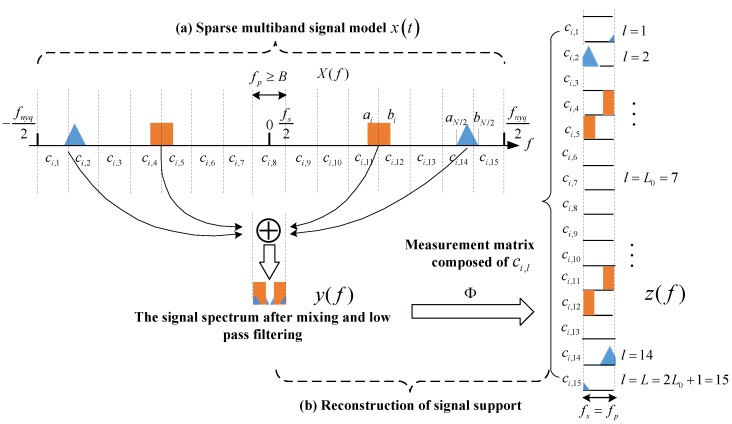
The principles of the MWC system [16,17]. (**a**) Sparse multiband signal model; (**b**) Reconstruction of signal support.

As depicted in Figure 1a, x(t) is the sparse multiband signal, which is a real signal and continuous in time. The spectrum of x(t) has at most N parts with energy in the whole frequency band. From [16], we know that the MWC contains a number of parallel sub-sampling channels, and each channel has the same hardware structure. The received continuous-time multiband signal is the input to the sub-sampling channels at the same time, and in each channel x(t) is multiplied by the periodic mixing signal $p_{i}(t)$ with a different mode, which can make the frequency spectrum of signal x(t) move to baseband. The $p_{i}(t)$ values of each channel are uncorrelated with each other. The period of $p_{i}(t)$ is $T_{p}=1/f_{p}$, where $f_{p}$ is the frequency of $p_{i}(t)$. M is used to show the number of random ±1 switches in a cycle. $Mf_{p}$ is defined as the switching frequency of mixed signals. The mixed signals pass through the low pass filter, whose cut-off frequency is $1/2T_{s}$. Finally, it passes through the ADC, whose sampling rate is $f_{s}=1/T_{s}$ and obtains the m groups low rate digital sampling sequences $y_{i}[n]$. 

Figure 1b shows the reconstruction process of the support of x(t). Assume that the number of bands is 4, $f_{s}=f_{p}$, and $f_{p}\geq B$. We divide the wideband spectrum into L spectrum slices, where $L=2L_{0}+1$. In order to ensure that the discrete Fourier transformation result of the sampling sequence contains all the components of the original signal spectrum X(f), $L_{0}$ must satisfy $L_{0}=\left\lfloor \left(f_{nyq}+f_{s}\right)/2f_{p}\right\rfloor -1$. After mixing and low pass filtering, the spectrum information of the original signal appears in the sampling interval $\left[-f_{s}/2,f_{s}/2\right]$, and the mixing coefficient of each spectrum slice is $c_{il}$, where l is the index of spectrum slices. According to the theory of CS, we can obtain the spectrum support of the multiband signal.

On analysis of the *i-*th channel, the Fourier series expansion of the random mixing function is:

(1) $$p_{i}(t)=\sum_{l=-\infty }^{\infty }c_{il}e^{j2\pi f_{p}lt}$$

In (1), $p_{i}(t)$ denotes a pseudo-random sequence of ±1, which is used as a mixing signal of the ith sampling channel; l is the index of the spectrum slice; and $c_{il}$ is the coefficient of the Fourier series expansion. The coefficient $c_{il}=d_{l}\sum_{k=0}^{L-1}\alpha_{ik}e^{-j\frac{2\pi }{L}lk}$, $\alpha_{ik}\in \{-1,+1\}$. When l=0, $d_{0}=1/L$, and when l≠0, $d_{l}=(1-e^{-j\frac{2\pi l}{L}})/j2\pi l$.

Then, after passing through the low pass filter, whose frequency characteristic is $H(f)=\{\begin{array}{l} 0\;\left|f\right|>f_{s}/2\\ 1\;\left|f\right|\leq f_{s}/2 \end{array}$, the relationship between the DTFT (Discrete Time Fourier Transform) of $y_{i}[n]$ is obtained by sampling, and the Fourier transform X(f) of x(t) is:

(2) $$Y_{i}(e^{j2\pi fT_{s}})=\sum_{l=-L_{0}}^{L_{0}}c_{i}{}_{l}X(f-lf_{p})$$

In (2), $f\in [-f_{s}/2,f_{s}/2]$, and $L_{0}$ is the smallest integer that makes $L=2L_{0}+1\geq f_{nyq}/f_{p}$. Equation (2) shows that the spectrum of the output sequence is changed into the shift weighted sum of the original signal spectrum with $f_{p}$ as its step, and it is intercepted into $f_{s}$ wide spectral fragments by the low pass filter. If $Y_{i}(e^{j2\pi fT_{s}})$ is considered as the *i-*th component of the m dimensional column vector y(f), and $X(f-lf_{p})$ as the *l-*th component of the $2L_{0}+1$ dimensional column vector z(f), then (2) can be expressed as:

(3) $$y(f)=\Phi z(f),\;f\in [-f_{s}/2,f_{s}/2]$$

In (3), Φ is an m×L matrix, $\Phi_{i}{}_{l}=c_{i,-l}=c_{il}*$, 1≤i≤m, and m<L. If the Inverse Discrete Time Fourier Transform (IDTFT) is performed on both ends of Equation (3), we can get the corresponding relationship between the sequence $Z[n]={\left[z_{1}[n],z_{2}[n],\ldots ,z_{L}[n]\right]}^{T}$ and the sampling data $Y[n]={\left[y_{1}[n],y_{2}[n],\ldots ,y_{m}[n]\right]}^{T}$; that is:

(4) Y[n]=ΦZ[n]

For any frequency $f\in [-f_{s}/2,f_{s}/2]$, (4) is a typical compressed sensing problem: when a measured matrix Y and an observation matrix Φ are known, a sparse vector Z can be recovered. In practical applications, the noise is included in Y.

In detail, the classic CS problem is that a Single Measurement Vector (SMV) is known to recover a single unknown sparse vector; that is, an SMV problem. However, in the application of the MWC, multiple measurement vectors are generated due to the parallel sampling structure of the MWC. Each measurement vector corresponds to an unknown signal vector, which is sparse and has a common support set. The multiple sparse vectors need to be simultaneously recovered under the condition that multiple measurement vectors are known. In summary, the reconstruction problem with such a joint sparse structure is called Multiple Measurement Vectors (MMV) problem [32,33], which can be expressed as the matrix. Therefore, it also can be considered that the MMV problem is composed of multiple SMV problems, and its essence is to achieve simultaneous recovery of a series of sparse vectors. As a result, paper [21] gets a one-dimensional vector after projecting Y subtly, and then reconstructs the signal support band by using the compressed sensing technique. Furthermore, paper [16] realizes the reconstruction by building a Continuous to Finite (CTF) module. Similar to the tensor completion problem [34,35], the observation matrix in the CTF module also has a low-rank property. 

In addition, from the MMV problem we can introduce the concepts of the joint support set and joint sparsity order.

**Definition** **1 (Joint Support Set) [36].** *Given a matrix $W=\left[w_{1},w_{2},\ldots ,w_{i},\ldots ,w_{n}\right]$, assuming that each column vector $w_{i}$ of the matrix W is sparse, and W has only a small number of common non-zero rows, then its joint support set can be expressed as follows:*

(5) $$\mathrm{supp}\left(W\right)=\underset{i\in \left\{1,2,\ldots ,n\right\}}{\cup }\mathrm{supp}\left(w_{i}\right)$$

**Definition** **2 (Joint Sparsity Order) [36,37].** *If the potential of supp(W) satisfies |supp(W)|≤K, then W is said to be joint K sparse, or we can also say that the joint sparsity order of W is K.*

**Remark** **1.** *In Definition 1, supp(W) is the union of the support of sparse vectors. From the perspective of the matrix, it can be understood as a set of index values of non-zero rows of unknown matrix W. In fact, the joint sparse model (JSM) generated by the MWC structure belongs to the second model in [37], namely JSM-2.*

### 2.2. System Model

In a Cognitive Radio Network (CRN), there are primary users and secondary users. Primary users (PUs) are the licensed users, also called legitimate/authorized users. PUs have the license to operate in the specified frequency band to access the primary base station (BS), which should not be affected by the operations of any other unlicensed users. Secondary users (SUs) are unlicensed users without a spectrum license, also referred to as “CR users,” or as “CRs” for brevity. SUs need to continuously monitor the activities of the licensed users to find the spectrum holes, which is also called spectrum sensing, and then look for opportunistic access to both the licensed and unlicensed spectrum band. Obviously, SUs are allowed to operate only if no interference is caused for licensed PUs [38,39].

**Figure 2:**
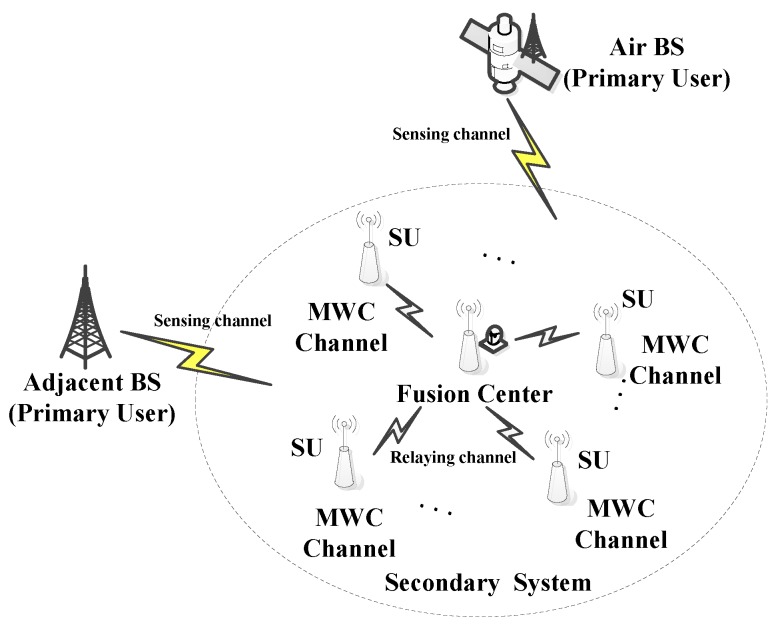
System model.

Because spectrum sensing is done in a very wide frequency range, we consider a CR network with m SUs, one FC, and several base stations (BS), in which the FC also contains a MWC sub-sampling channel, as depicted in Figure 2. The multiple secondary user nodes and several primary users are randomly distributed. The primary user can be a nearby signal transmitting base station, or an air-sky transmitting base station that is composed of an airship or a satellite. The signals transmitted by primary users occupy a fixed authorized frequency band. The spectrum sensing channel is a wideband link between the PUs and the SUs. In the secondary system, each sensing node samples the PU signal over the spectrum sensing channel in a compressed manner. Each sensing node is considered as one compressed sampling channel within the parallel MWC hardware sampling structure. The channel between a sensing node and FC is considered the relaying channel. Firstly, SUs transmit the compressed sampling data to the FC through the relaying channel. Then, the FC performs wideband spectrum sensing on the compressed data received from the SUs and the compressed data sampled by itself. Finally, after the FC obtains signal spectral support within a very wide spectrum range, the FC will feedback the sensing results to the SUs.

In CR communications, the signal detected between extremely wide spectral ranges is usually considered a sparse multiband analog signal x(t), which contains N sub-bands with signal energy. The x(t) is shown in Figure 1a, whose frequency support resides in a union of k disjoint bands which are spread over a very wide spectrum range $\left[-f_{nyq}/2,f_{nyq}/2\right]$. The whole spectrum range is divided into L consecutive narrow band channels, with the bandwidth of each sub-band not exceeding B. The sub-band unions of x(t) and the maximum bandwidth B can be expressed as:

(6) $$P_{N}=\overset{N/2}{\underset{i=1}{\cup }}\left\{\left(a_{i},b_{i}\right)\cup \left(-b_{i},-a_{i}\right)\right\},\;B=\underset{i}{\max}\left(b_{i}-a_{i}\right)$$

If the sub-bands are marked as [1,…,L], the set of all occupied sub-bands $X_{i}(f)$ is the spectral support of the signal x(t), which is defined as Λ=supp(X(f)). |Λ| is the potential of spectral support. 

Considering the WCSS, the sensing performance may degrade for several reasons, such as multipath fading, phase shift, and noise uncertainty. The paper by Xu et al. [30] verified that phase shift has no influence on the recovery of support. However, a change of the distance between the SU and FC will lead to a change of the multipath channel parameters, such as the signal delay spread. In order to simplify the problem, we only consider the different delay paths at the receiving end caused by the diffuse reflection of several adjacent SUs, which results in a typical frequency selective fading effect. By drawing lessons from the analysis method of Driessen’s paper [40], the coefficient of radio wave propagation loss $L_{j}(t_{d})$ of jth SU is calculated by:

(7) $$L_{j}(t_{d})=\frac{P_{R}(t=(r_{TS}+r_{SR})/c)}{P_{T}(t=0)}=\frac{\lambda^{2}}{{\left(4\pi \right)}^{3}}\int_{A}\frac{\sigma_{j}{}^{0}}{r_{TS}{}^{2}r_{SR}{}^{2}}dA_{j}\approx \frac{\lambda^{2}}{{\left(4\pi \right)}^{3}}\sum_{i}\frac{\sigma_{ij}{}^{0}dA_{ij}}{r_{TS}{}^{2}r_{SR}{}^{2}}$$

The derivation of Equation (7) is given in Appendix A. In Equation (7), $P_{R}(t=(r_{TS}+r_{SR})/c)$ denotes the received power when $t=(r_{TS}+r_{SR})/c$, $P_{T}(t=0)$ denotes the transmission power at t = 0, λ is the wavelength, $r_{TS}$ is the distance between the transmitter and the scatterer, $r_{SR}$ is the distance between the receiver and the scatterer, $r_{TR}$ is the distance between the transmitter and the receiver, $\sigma^{0}$ denotes the root mean square of slope on any small diffuse reflection area dA, c is the signal transmission speed, and A indicates the area of effective diffuse reflection. $t_{d}$ is given as:

(8) $$t_{d}=(r_{TS}+r_{SR}-r_{TR})/c$$

Thus, in the FC, the received signal from -th SU can be represented as:

(9) $$x^{\prime }{}_{j}(t)=L_{j}(t_{d})x_{j}(t)$$

### 2.3. Problem Statement

As previously mentioned, the FC obtains sub-Nyquist sampling signals from SUs, and then the FC reconstructs the frequency support of x(t). However, if the reconstruction of the spectral support is conducted by using a traditional CS recovery algorithm, some problems remain such as the need for too many sampling channels, poor sensing accuracy in the presence of transmission interference, and unknown prior information in practical sensing conditions.

In (4), considering the practical time-domain sampling process, the obtained matrix Y(n) must be finite dimensional, and Z(n) is joint sparse. Therefore, (4) is a typical MMV problem [41,42], which can be transformed into the solution of the constrained optimization problem:

(10) min I(Z(n)) s.t. Y(n)=ΦZ(n)

In (10), I(Z(n)) is the joint sparsity order of Z(n). Mishali has proved that the MWC can achieve a high recovery success probability when m>4Nlog(L/2N) [16]. However, there is still a big gap between m and the theoretical lower limit (m=2N) [16], and m is limited by the number of SUs in this paper. Furthermore, in earlier research [19,20,21,22,43], the reconstruction schemes of spectral support needed to know the signal sparsity order, which is very difficult to achieve in practice. In [20], the decision condition of support is given by ${\left\|{\tilde{Z}}^{i\to }\right\|}_{2}\geq \varepsilon$, and the threshold ε is a predetermined fixed value. However, in practice, ε is closely related to the noise intensity. In addition, in the presence of noise interference and transmission loss, it is difficult to achieve a high success recovery probability of support with a single support selection scheme in the existing literature.

In addition, it has been shown in [30] that if the single support selection strategy is used in the FC, the number of sub-Nyquist sampling channels will be far greater than the theoretical lower bound, and the sensing performance becomes terrible under low SNR or low loss gain. Thus, in order to improve sensing performance, the proposed PSS-SaDMWC scheme uses the progressive support selection strategy to reconstruct the spectral support. 

## 3. Proposed PSS-SaDMWC Scheme

In this section, based on SVD, the estimation methods of noise intensity and sparsity order are given. Then, we discuss the effect of transmission loss on the PSS-SaDMWC scheme. Finally, the pseudo-code description of the PSS-SaDMWC scheme is given, and the convergence of the scheme is proved.

### 3.1. Preprocessing of PSS-SaDMWC Scheme

#### 3.1.1. Estimation of Noise Intensity

In the FC, due to the influence of noise intensity changes, the decision threshold cannot be set to a fixed value. Inspired by Liu [44], we used the SVD to estimate noise intensity. We performed singular value decomposition on Y to obtain the singular values vector of Y; that is, $\sum_{Y}=\left(\sigma_{1},\sigma_{2},\ldots ,\sigma_{K},\ldots \sigma_{m}\right)$. Y is composed of real signal and noise. Similarly, we can also obtain the singular values vector of the noise and the singular values vector of the real signal, denoted as $\sum_{N}=\left(\sigma_{n1},\sigma_{n2},\ldots ,\sigma_{nK},\ldots \sigma_{nm}\right)$ and $\sum_{S}=\left(\sigma_{s1},\sigma_{s2},\ldots ,\sigma_{sK},\ldots \sigma_{sm}\right)$ respectively, where $\sigma_{i}$ is the *i*th singular value. Obviously, in practical applications, $\sum_{Y}$ is known, but $\sum_{N}$ and $\sum_{S}$ are unknown. In the next paragraph, we use the given real signal data and the given noise data in the experiments to help the analysis.

**Figure 3:**
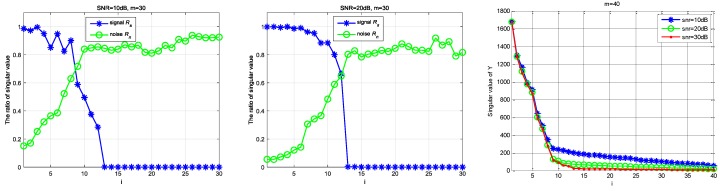
The contribution of the singular value of the signal and noise to total singular value.

**Figure 4:**
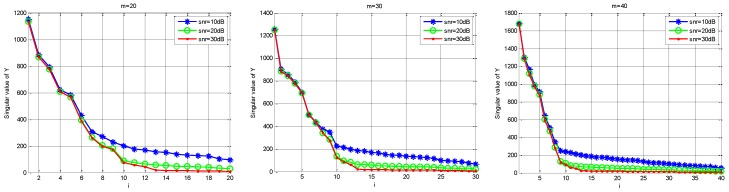
The comparison of the singular value of Y under different SNR environments.

**Figure 5:**
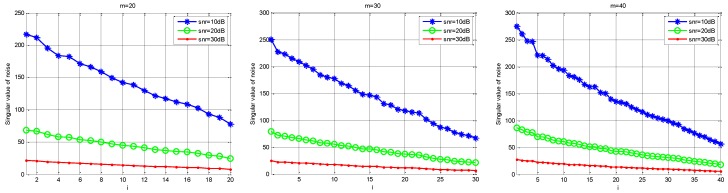
The distribution of the noise singular value.

Firstly, we define $R_{n}$ and $R_{s}$. $R_{n}$ is the contribution of the noise singular value to the singular value of Y; that is, $R_{n}=\left(\sigma_{n1}/\sigma_{1},\sigma_{n2}/\sigma_{2},\ldots ,\sigma_{nK}/\sigma_{K},\ldots ,\sigma_{nm}/\sigma_{m}\right)$. $R_{s}$ is the contribution of the singular value of the real signal to the singular value of Y; that is, $R_{s}=\left(\sigma_{s1}/\sigma_{1},\sigma_{s2}/\sigma_{2},\ldots ,\sigma_{sK}/\sigma_{K},\ldots ,\sigma_{sm}/\sigma_{m}\right)$. Under different SNR levels, the relationship between $R_{s}$ and $R_{n}$ is shown in Figure 3. Next, for the received under-sampling signal Y, Figure 4 shows the distribution of singular values under different noise intensities and different sampling channel numbers. Finally, Figure 5 shows that the distribution of the real noise singular values is basically linear. In Figure 3 and Figure 4, the loss gain is set to 0.9.

As can be seen from Figure 3 and Figure 4, the tail of the singular value is mainly determined by noise, the distribution of which presents a linear feature, and the energy of the signal is concentrated in the first K singular values, where K is the sparsity order of the signal. Furthermore, the initial singular values show little change no matter how much the SNR and channel number have changed. However, the tail of the singular values is chiefly influenced by noise intensity because the contribution of the original signal is small. As shown in Figure 4, the tail singular values fluctuate a great deal under different SNR levels, and the tail distribution of the singular values is basically linear. Therefore, we can assume that the relationship is linear between the mean value $M_{P}$ of the P tail singular values and the real noise intensity σ, as in:

(11) $$M_{P}=\alpha \sigma +\lambda$$

In (11), α and λ are the unknown parameters. In order to solve the unknown parameters and estimate the noise intensity, we add the known Gauss white noise into the received signal at the fusion center. Then, we can establish a set of equations, as follows:

(12) $$M_{P1}=\alpha \sqrt{\sigma^{2}+\sigma_{1}{}^{2}}+\lambda$$

(13) $$M_{P2}=\alpha \sqrt{\sigma^{2}+\sigma_{2}{}^{2}}+\lambda$$

The proof of (12) is given in [44]. Because the $M_{P1}$, $M_{P2}$, $\sigma_{1}$ and $\sigma_{2}$ are known, by using Equations (11)–(13), we can get the value of α, λ and σ. The estimated σ is expressed as:

(14) $$\begin{array}{cc} \hat{\sigma }= & \frac{\sqrt{\left(M_{P1}-M_{P2}\right)/\left(\sigma_{1}{}^{2}/\left(M_{P1}-M_{P}\right)-\sigma_{2}{}^{2}/\left(M_{P2}-M_{P}\right)\right)}}{2\times \left(M_{P1}-M_{P}\right)}\sigma_{1}{}^{2}-\\ & \frac{\left(M_{P1}-M_{P}\right)}{2\times \sqrt{\left(M_{P1}-M_{P2}\right)/\left(\left(\sigma_{1}{}^{2}/\left(M_{P1}-M_{P}\right)-\sigma_{2}{}^{2}/\left(M_{P2}-M_{P}\right)\right)\right)}} \end{array}$$

**Remark** **2.** *The noise intensity estimation is mainly based on two aspects: (1) the singular values of the signal tail are mainly affected by the noise energy; (2) the singular values of noise present linear characteristics. However, in fact, the tail singular values of the signal contain very little signal energy, and the linear characteristic of the noise singular values is not perfect. These will lead to some deviations between the estimated value and the actual result. In practice, we can select the appropriate number of tail singular values to reduce the estimation error*.

#### 3.1.2. Estimation of Sparsity Order

The sparsity order estimation can be implemented using CS theory in combination with other statistical learning methods, such as Gaussian mixture models [45]. However, because some of the results in Section 3.1.1 can be obtained directly, we used the SVD combined with the gradient and difference methods to estimate signal sparsity order in this section.

If 30% tail singular values are used as noise singular values to perform linear fitting, we can get the estimated noise singular values ${\hat{\Sigma }}_{n}=diag({\hat{s}}_{n1},\ldots ,{\hat{s}}_{ni},\ldots ,{\hat{s}}_{nm})$, where ${\hat{s}}_{ni}$ is the *i*th estimated noise singular value. The contribution of noise singular values to the singular values of *Y* is calculated by Equation (15):

(15) $${\hat{R}}_{n}={\hat{\Sigma }}_{n}./\Sigma =\overset{m}{\underset{i=1}{\cup }}({\hat{s}}_{ni}/s_{i})$$

(16) $$G_{R_{n}}=abs\left(\mathrm{gradient}({\hat{R}}_{n},1)\right)\;D_{R_{n}}=diff(G_{R_{n}})$$

**Figure 6:**
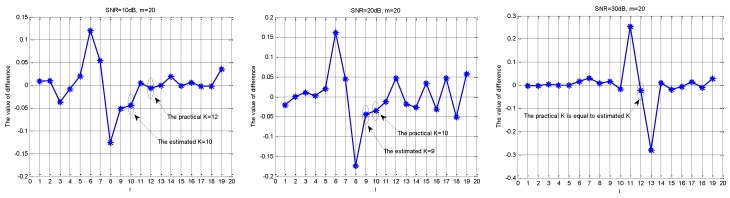
The estimation of the sparsity order under different SNR levels.

$G_{R_{n}}$ and $D_{R_{n}}$ are defined in (16), where abs() is a function which is used to find absolute values. If the noise intensity is strong, $G_{R_{n}}$ is first obtained by performing the gradient operation on ${\hat{R}}_{n}$, and then $D_{R_{n}}$ is obtained by performing the difference operation on $G_{R_{n}}$. The calculation method of $G_{R_{n}}$ and $D_{R_{n}}$ is shown in (16). The results of the operation are in ascending order. The position of the minimum value in $D_{R_{n}}$ plus 1 is the estimated sparsity order $\hat{K}$. If the sampling channel number is close to the theoretical lower limit, $\hat{K}$ needs to add one adjustable parameter *e*, where the empirical value e is usually set to 1. Figure 6 is a sketch map of the sparsity order estimation under different SNR levels. 

If the noise is weak, the signal energy is dominant. The sparsity order can be estimated directly by using the singular values of Y. Firstly, all singular values are shifted to the left one time, and the last empty position is filled by $s_{m}$, represented as $\Sigma_{a}=diag(s_{2},\ldots ,s_{i},\ldots ,s_{m},s_{m})$. Then, R is calculated by (16), and *R* is in descending order. The index of the maximum value in *R* is the estimated sparsity order $\hat{K}$. Although $\hat{K}$ has some deviations, due to adopting the progressive support selection strategy the deviation has no impact on the success rate of reconstruction:

(17) $$R=\Sigma_{a}./\Sigma \;$$

**Remark** **3.** *Since the noise singular values are obtained by linear fitting, the estimation error is directly related to the signal-to-noise ratio. If the signal-to-noise ratio is too small, the estimation of the noise singular values will be biased. Although these deviations can be corrected by the empirical correction parameter, they still affect the estimation accuracy of the sparsity order. In practice, we will list an error correction parameter table and choose appropriate correction parameters for different actual scenarios*.

### 3.2. The Influence of Propagation Loss on Support Reconstruction

When the sub-Nyquist sampling data is transmitted to the fusion center by the CR node, transmission loss is inevitable. Thus, according to the conclusion in Section 2.2, Equation (2) can be expressed as:

(18) $$Y_{i}(e^{j2\pi fT_{s}})=\sum_{l=-L_{0}}^{L_{0}}L_{j}(t_{d})e^{j\theta_{j}}c_{i}{}_{l}X(f-lf_{p})\;f\in [-f_{s}/2,f_{s}/2]$$

Firstly, from Xu’s research [30], we can draw the conclusion that phase shift $\theta_{j}$ in the jth path has no effect on the spectral support recovery. However, in the practical networks, we have to investigate the influence of propagation loss on the recovery success rate of support. In the presence of observation noise and channel fading, the optimization problem with the equality constraints of (10) can be relaxed as a constrained inequality optimization problem:

(19) $$\underset{z}{\min}{\left\|Z(n)\right\|}_{1}\;s.t.\;{\left\|Y(n)-\Phi Z(n)\right\|}_{2}\leq \zeta$$

where ζ≥0 is a permissible error disturbance. Here, $\Phi_{i}{}_{l}=L_{ji}(t_{d})c_{i,-l}=L_{ji}(t_{d})c_{il}*$. In addition, from Theorem 1, we also can conclude that (19) is convergent.

**Theorem** **1.** *Let $Z\in {ℜ}^{n}$, $\Phi \in {ℜ}^{m\times L}$, for a positive constant C, if m satisfies:*

(20) $$m\geq C\mu^{2}\left(\Phi \right)K\log\left(L/\delta \right)$$

*where m is the number of parallel channels, $\mu \left(\Phi \right)=\underset{j\neq i}{\max}\frac{\left|\langle \phi_{i},\phi_{j}\rangle \right|}{{\left\|\phi_{i}\right\|}_{2}{\left\|\phi_{j}\right\|}_{2}}$ is the maximum absolute value of the cross-correlation between the different columns in Φ, and $\mu \left(\Phi \right)\in \left\lfloor \sqrt{L-m/m\left(L-1\right)},1\right\rfloor$ [46]. Then, the solution of (19) can be obtained by the probability precision of 1−δ. In other words, the high-dimensional discrete sparse signal Z can be reconstructed from the low dimensional sampling signal Y with the probability of 1−δ.*

**Proof.**  The proof of Theorem 1 can be found in [47]. □

**Figure 7:**
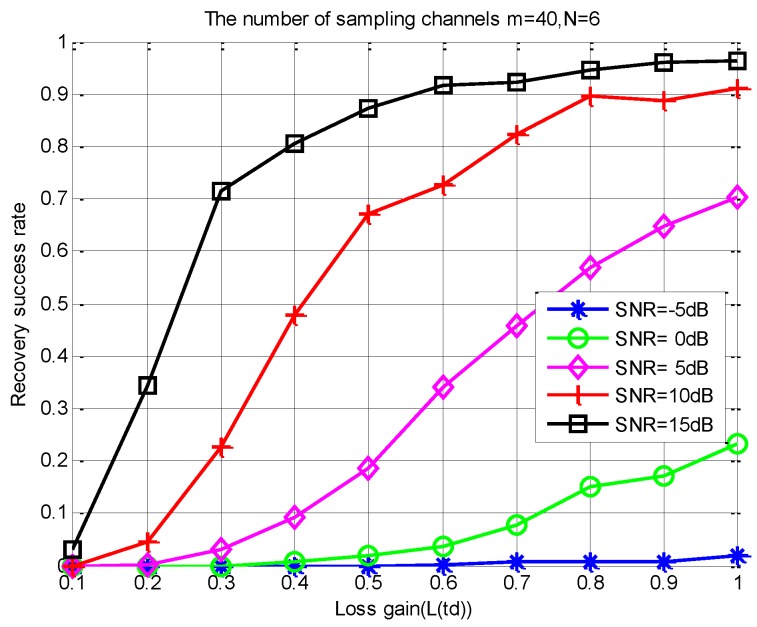
Influence of loss gain on recovery accuracy under different SNR levels.

**Figure 8:**
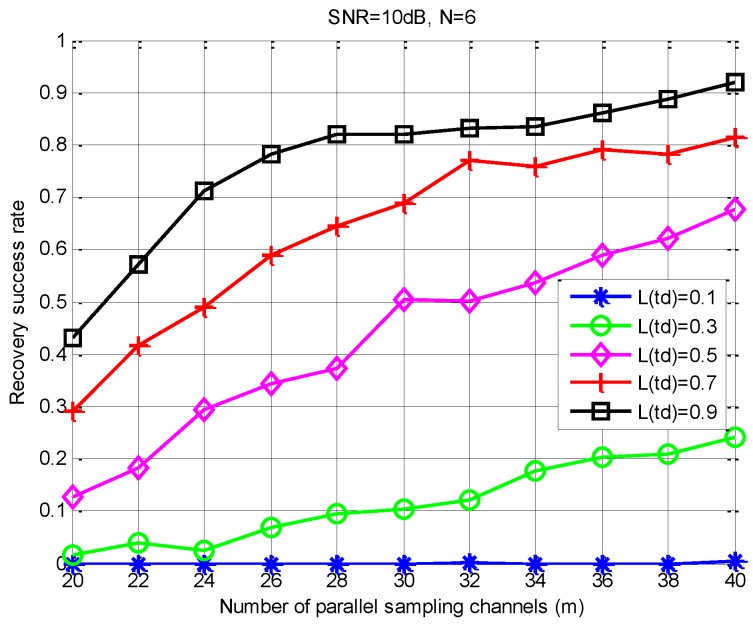
Influence of the sampling channels number on recovery accuracy under different loss gains.

**Remark** **4.** *It is worthwhile to note that, in (20), an important constraint relationship is established between the observation matrix Φ, the number of measurements m, the spectral slice number L, and the sparsity order K. In addition, when the correlation between the column vectors in Φ is smaller, the number of measurement samples required is less. From (20), we can get an acceptable loss gain through the constraint of the sampling channels number. For a given N, we can get an intuitive relationship from Figure 7 and Figure 8 between m, the SNR level, the successful recovery probability, and the loss gain $L(t_{d})$. In Figure 7 and Figure 8, we use the S-MUSIC scheme [43] based on a single support selection strategy.*

According to (18) and Theorem 1, loss gain has an influence on the correlation between the column vectors of the observation matrix. From Figure 8, this correlation directly affects the number of sampling channels m. Moreover, m directly corresponds to the number of SUs involved in spectrum sensing. Thus, in order to obtain a high support reconstructed probability, the transmission loss is too large to lead to the need for too many sampling channels, which is impossible in real CR communications. Therefore, we need a suitable and acceptable m to ensure a high recovery success rate of spectral support. 

### 3.3. Support Selection Strategy Based on Progressive Operation

From Section 3.2, we know that the successful recovery rate is very low from using the single selection strategy when the SNR is low and the channel loss is large. In order to further illustrate the problem, we conduct 100 experiments separately using the RPMB scheme [20] and S-MUSIC scheme [43]. In each experiment, different multi-band signals were randomly generated as sparse signal sources. Meanwhile, each experiment takes 20 iterations, and each gets a signal support. If the correct spectral support exists in the 20 supports of the sets, we investigate the ability of the two methods to extract the correct spectral support in the 100 experiments, respectively. The results are shown in Table 2. 

**Figure 9:**
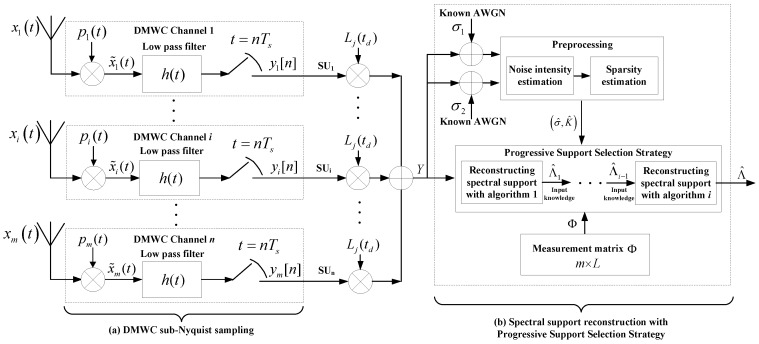
The structure of the PSS-SaDMWC scheme. (**a**) DMWC sub-Nyquist sampling; (**b**) Spectral support reconstruction with progressive support selection strategy.

The above analysis shows that the extraction ability is weak in the case of the correct spectral support in the vector set, which leads to a very low successful recovery rate. However, if we combine the two algorithms for progressive extraction, we can get good results. Figure 9 demonstrates the system structure of the PSS-SaDMWC scheme. 

To reduce the time complexity, the PSS-SaDMWC scheme generally only needs two to four levels for progressive reconstruction. The PSS-SaDMWC scheme, based on the progressive support selection strategy and preprocessing methods, is summarized in Algorithm 1. 

**Algorithm 1.** The description of the PSS-SaDMWC scheme.**Parameters**: x(t), N, B, $f_{nyq}$, L, $f_{s}$, $f_{p}$, $r_{SR}$, $r_{TS}$, $\sigma_{1}$, and $\sigma_{2}$. **Initializations**: ${\hat{\Lambda }}_{0}=\emptyset$. **I. DMWC compressed sampling**(a)Deliver the sub-Nyquist observation by (2).(b)Solve (7) to obtain the loss coefficient.(c)Construct $Y(e^{j2\pi fT_{s}})$ according to (18), and get the observation matrix Φ.**II. Pretreatment process**(a)Add AWGN $\sigma_{1}$ to Y, we can get (12). Then, add AWGN $\sigma_{2}$ to Y, we get (13).(b)Solve (14) to obtain $\hat{\sigma }$.(c)Get $\hat{K}$ by (16) and (17).**III. Reconstruction of spectral support based on progressive support selection**(a)Construct an initial weight vector: $W_{0}=\left\{w_{0,1},w_{0,2},\ldots ,w_{0,i},\ldots ,w_{0,2\hat{K}}\right\}=\left\{0\right\}$.(b)According to [48], we can get $\left[W_{1},{\hat{\Lambda }}_{1}\right]$ = *Reconstruction_algorithm_1*(Y, Φ, $\hat{\sigma }$, $\hat{K}$, ${\hat{\Lambda }}_{0}$, $W_{0}$).(c)$\left[W_{2},{\hat{\Lambda }}_{2}\right]$ = *Reconstruction_algorithm_2*(Y, Φ, $\hat{\sigma }$, $\hat{K}$, ${\hat{\Lambda }}_{1}$, $W_{1}$).… …(d)$\left[W_{n},{\hat{\Lambda }}_{n}\right]$ = *Reconstruction_algorithm_n*(Y, Φ, $\hat{\sigma }$, $\hat{K}$, ${\hat{\Lambda }}_{n-1}$,, $W_{n-1}$).**Output:**${\hat{\Lambda }}_{n}$.

In Algorithm 1, $w_{i,j}$ represents the importance of the *j*th support element in the *i-*th reconstruction. The progressive idea is included in Algorithm 1. The reconstruction algorithms in Algorithm 1 are actually an improvement on the existing reconstruction algorithms. The improved reconstruction algorithms do not need to know the sparsity order of the signal in advance, in which the decision threshold can also be adaptively adjusted by the estimated noise intensity, and the idea of progressive support selection is also incorporated into the improved reconstruction algorithm. 

The concrete implementation of the progressive idea is embodied in each support reconstruction algorithm, the pseudo codes of which are expressed as follows (Algorithm 2):

**Algorithm2.** The *i*th support reconstruction with the progressive idea.**Input parameters**: Y, Φ, $\hat{\sigma }$, $\hat{K}$, ${\hat{\Lambda }}_{i-1}$, $W_{i-1}$. **Obtain progressive spectral support:**(a)$2\hat{K}$ elements of spectral support are obtained by using input parameters (Y, Φ, $\hat{\sigma }$, $\hat{K}$) and *i*th reconstruction Algorithm, named $\dot{\Lambda }$.(b)j = 0;while $j<2\hat{K}$  if ($\dot{\Lambda }\left(j\right)\in {\hat{\Lambda }}_{i-1}$)$pos=find\left({\hat{\Lambda }}_{i-1}==\dot{\Lambda }\left(j\right)\right)$;    Update the weights: $W_{i}\left(j\right)=W_{i-1}\left(pos\right)+0.1$, and delete $W_{i-1}\left(pos\right)$ and ${\hat{\Lambda }}_{i-1}\left(pos\right)$    from vector $W_{i-1}$ and vector ${\hat{\Lambda }}_{i-1}$, respectively.  else  $W_{i}\left(j\right)=0$;  end  j = j+1;end(c)After the loop, obtain a new $W_{i-1}$ vector and a new ${\hat{\Lambda }}_{i-1}$ vector, named $W^{\prime }{}_{i-1}$ and $\hat{\Lambda }{{}^{\prime }}_{i-1}$.(d)$W_{temp}=sort(W_{i}\cup W{{}^{\prime }}_{i-1},\;{}^{\prime }descend{}^{\prime })$, and $W_{i}=W_{temp}(1:2\hat{K})$.(e)According to $W_{i}$, $2\hat{K}$ support elements corresponding to the weights are selected from vector $\dot{\Lambda }\cup \hat{\Lambda }{{}^{\prime }}_{i-1}$ to form a new spectral support ${\hat{\Lambda }}_{i}$.**Output:**${\hat{\Lambda }}_{i}$ and $W_{i}$.

## 4. Performance Evaluation and Analysis

### 4.1. Design Example and Performance Metrics

Firstly, we simulated the scheme using *sinc* signal contaminated by white Gaussian noise. The sparse multiband analog signal with noise is generated by (21), which consists of three pairs of bands (total N=6):

(21) $$x(t)=\sum_{i=1}^{N/2}\sqrt{E_{i}B_{i}}\sin c\left(B_{i}\left(t-\tau_{i}\right)\right)\cos\left(2\pi f_{i}\left(t-\tau_{i}\right)\right)+n(t)$$

where sinc(x)=sin(πx)/πx, x(t) is a multi-band signal, and n(t) is a white Gaussian noise. For the multi-band signal, the carriers $f_{i}$ are chosen uniformly and randomly in $\left[-f_{nyq}/2,f_{nyq}/2\right]$. Table 3 lists the simulation parameters and their meanings. The PSS-SaDMWC scheme performs the process as Algorithm 1. The reconstruction algorithms of the PSS-SaDMWC in numerical experiments include an improved SwSOMP_MWC and an improved S-MUSIC_MWC.

Next, the simulations are performed with MATLAB to evaluate the performance of the proposed scheme against the existing schemes, and the following procedure was repeated 500 times to calculate the recovery success probability:

(1) Generate the mixing signal $p_{i}\left(t\right)$ randomly.
(2) Generate the carrier frequency $f_{i}$ uniformly and randomly in $\left[-f_{nyq}/2,f_{nyq}/2\right]$.
(3) Generate new *sinc* signal according to $f_{i}$.
(4) Estimate the spectral support using SOMP_DMWC [30], SwSOMP_DMWC [48], ReMBo_DMWC [21], RPMB_DMWC [20], S-MUSIC_DMWC [43], and PSS-SaDMWC, respectively, and determine whether the support is successfully recovered.

Finally, to evaluate the efficiency and reconstruction performance of proposed scheme (see Figure 9), we chose the following four performance metrics: (1) the recovery success rate of support; (2) the required minimum SUs number and minimum sampling rate; (3) the time-varying support recovery ability; (4) finding an acceptable $L_{j}(t_{d})$ under different SNR levels; and (5) a discussion of time complexity.

### 4.2. Simulation Results and Analysis

(1) The recovery success rate of support: When $P_{f}$ is less than or equal to the upper bound, we refer to the successful recovery criteria in [16]; that is, when the estimated support $\hat{\Lambda }$ and the actual support Λ meet the constraint condition given by Equation (22), where $\hat{\Lambda }⊇\Lambda$, and $\Phi_{↓\hat{\Lambda }}$ is with full column rank, it is considered a successful reconstruction. If the recovery success rate is more than 90%, it is considered a high probability reconstruction:

(22) $$\mathrm{success}\;s.t.\;\left(P_{f}\leq E_{upper}\&\&\hat{\Lambda }⊇\Lambda \&\&Rank(\Phi_{↓\hat{\Lambda }})={\left\|\hat{\Lambda }\right\|}_{0}\right)$$

(23) $$P_{f}=\left({\left\|\hat{\Lambda }\right\|}_{0}-{\left\|\Lambda \right\|}_{0}\right)/L$$

(24) $$E_{upper}={\left\|\Lambda \right\|}_{0}/L$$

where ${\left\|\hat{\Lambda }\right\|}_{0}$ is the potential of the reconstructed spectral support; that is, the length of support $\hat{\Lambda }$; ${\left\|\Lambda \right\|}_{0}$ is the potential of the real spectral support; L is the number of spectrum slices; $P_{f}$ is the false alarm probability; and $E_{upper}$ is an acceptable upper bound of $P_{f}$—that is to say, in L spectrum slices, there are at most ${\left\|\Lambda \right\|}_{0}$ false alarm sub-bands allowed. 

**Figure 10:**
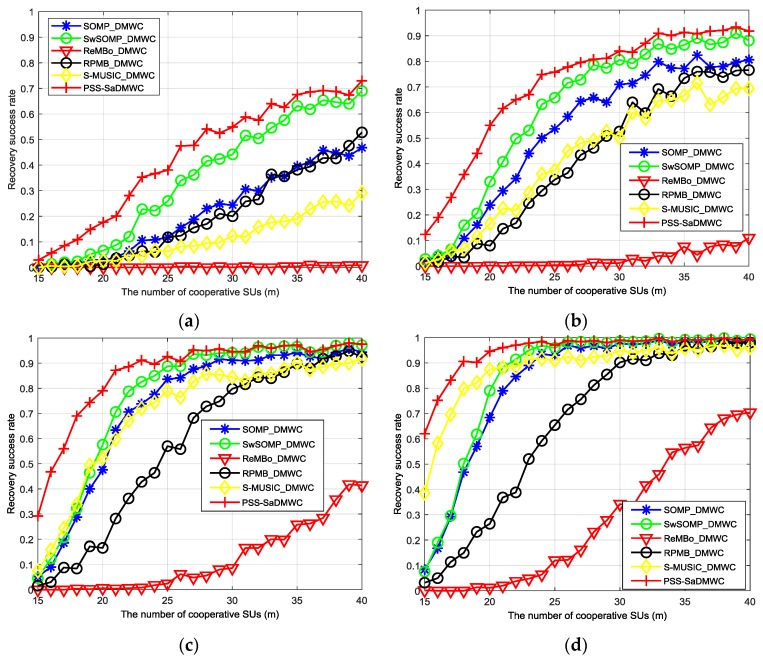

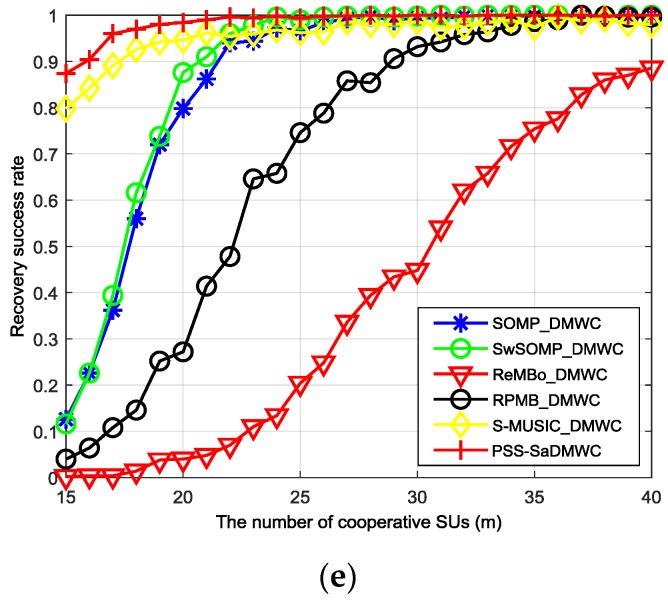
Reconstruction performance comparison between the PSS-SaDMWC and other existing single selection schemes. (**a**) SNR = 0 dB; (**b**) SNR = 5 dB; (**c**) SNR = 10 dB; (**d**) SNR = 15 dB; (**e**) SNR = 20 dB.

Figure 10 shows the support recovery success rate of the proposed PSS-SaDMWC scheme in comparison with the other existing single support selection schemes, such as SOMP_DMWC, SwSOMP_DMWC, ReMBo_DMWC, RPMB_DMWC, and S-MUSIC_DMWC. Obviously, the PSS-SaDMWC scheme outperforms the other schemes on the whole. When the number of cooperative SUs is small, the PSS-SaDMWC scheme can improve the performance by 23%–34% compared with the SwSOMP_DMWC scheme, in the case of a lower SNR. Even when the SNR is 15 dB or 20 dB, the PSS-SaDMWC scheme also can effectively increase the recovery success rate. Compared with the best single selection scheme, the maximum performance promoting rate is shown in Table 4.

**Figure 11:**
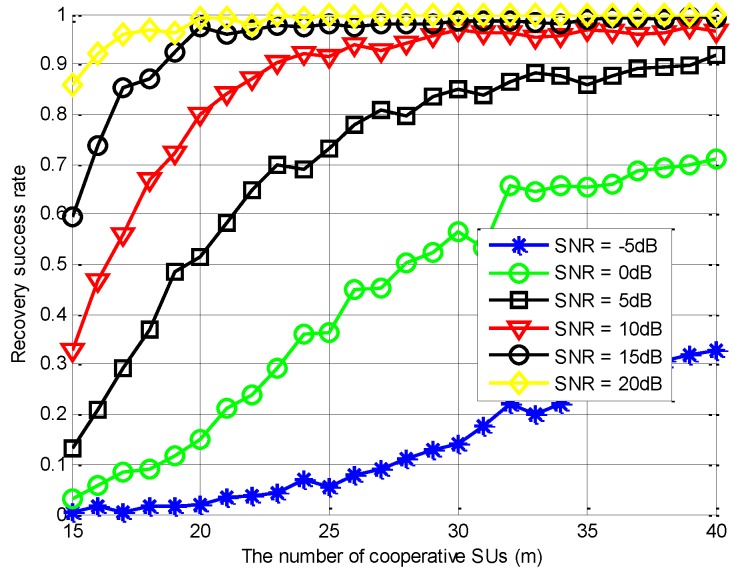
Performance comparison of the PSS-SaDMWC scheme under different SNR levels.

Subsequently, we change the SNR levels and evaluate the reconstruction performance with different m values, as Figure 11 shows. Specifically, when m is above 23 and the SNR is above 5 dB, the successful recovery rate improves steadily as m increases. When the SNR is less than 5 dB, more cooperative users are required to obtain high reconstruction probabilities. Finally, when SNR = 20 dB, the PSS-SaDMWC scheme can achieve a high probability reconstruction with the number of cooperative users close to the theoretical lower bound.

(2) The required minimum number of sampling channels and minimum sampling rate: In theory, Mishali’s paper [16] points out that the spectral support of the signal can be reconstructed as long as the number of sampling channels is satisfied by *m* ≥ 2*N*. In addition, the MWC can achieve sub-Nyquist sampling. The total sampling rate of MWC is $f_{\Sigma }=mf_{s}$. The theoretical minimum of the sampling rate for the multiband signal (that is, the Landau rate [21]) is defined as:

(25) $$M=2\sum_{i=1}^{N/2}\left(b_{i}-a_{i}\right)$$

where M is Landau rate, which is the sum of all sub-band frequency widths, and $\left(b_{i}-a_{i}\right)$ is the frequency width of the *i*th sub-band. Since the number of the SUs m and total sampling rate $f_{\Sigma }$ are directly related, the smaller the m is, the lower the cost of the system and the corresponding sampling rate.

As shown in Figure 10 and Table 5, under different SNRs, the number of hardware channels and the sampling rate of the PSS-SaDMWC scheme for the high probability reconstruction are smaller those that of the other single selection schemes. Thus, the proposed scheme in this paper can use fewer hardware channels and a lower sampling rate to achieve a high success rate of reconstruction. Obviously, the lower the number of hardware channels needed by the scheme, the more the system can save in costs; and the lower the sampling rate, the easier it is to implement the system hardware. 

**Figure 12:**
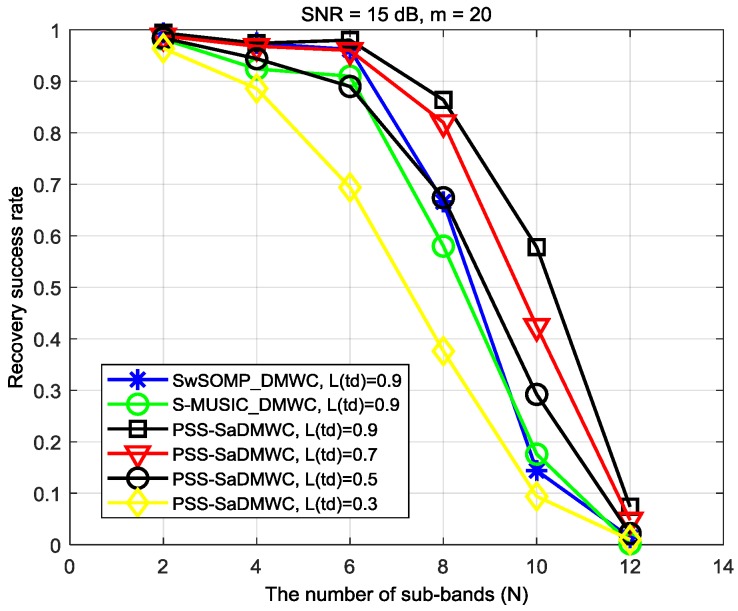
The support recovery ability under different numbers of sub-bands.

**Figure 13:**
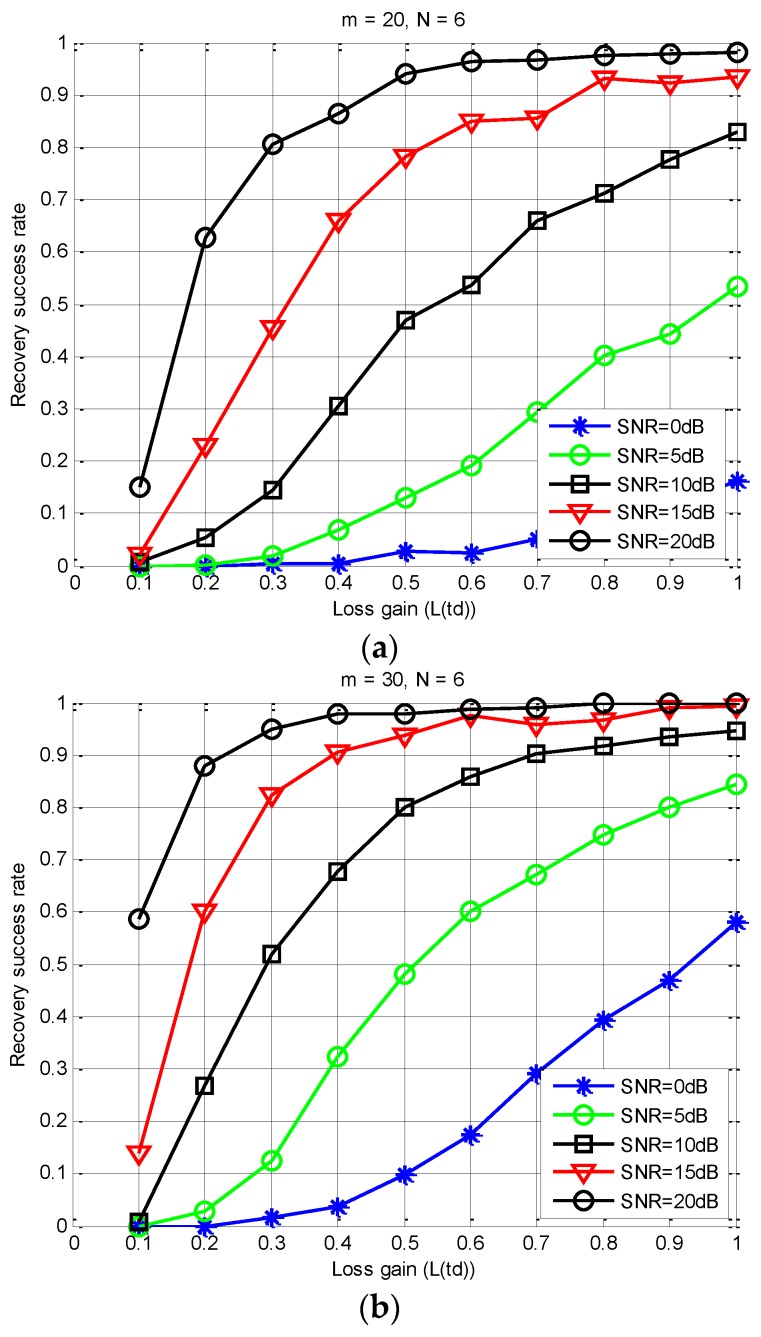
Recovery success rate versus the loss gain. (**a**) m = 20, N = 6; (**b**) m = 30, N = 6.

(3) Time-varying support recovery ability: From [41], if more sub-bands can be reconstructed by the scheme, the reconstruction ability of the scheme is stronger. Obviously, the increase of N leads to the increase of the signal sparsity order. As shown in Figure 12, the reconstruction performance of the PSS-SaDMWC is significantly better than that of SwSOMP_DMWC and S-MUSIC_DMWC under the same conditions. Specifically, when N=8, the reconstruction performance is improved by 20% and 27%, respectively, compared to that of SwSOMP_DMWC and S-MUSIC_DMWC. In addition, when N=10, the reconstruction performance is improved by 41% and 39%, respectively. Nevertheless, when the signal is not sparse enough, the recovery ability of all schemes will decrease sharply. Thus, when spectral support Λ is time-varying, the PSS-SaDMWC can dynamically increase the amount of cooperative SUs to obtain a stable support recovery ability. That is to say, the number of sampling channels can be flexibly added when N is large or the transmission loss is large.

(4) Finding an acceptable $L_{j}(t_{d})$ under different SNR levels: Figure 10 shows that the proposed scheme can significantly improve the sensing accuracy. However, Theorem 1 indicates that the decrease of the loss gain increases the correlation between the columns of the observed matrix, which leads to a poor sensing performance. The average recovery success rate versus the loss gain under different instantaneous SNR conditions is shown in Figure 13. Obviously, in practical applications, we hope to find a minimum transmission loss that must be satisfied according to a given sensing accuracy. Then, the constrained distance between the SUs and FC can be estimated by (7). Therefore, for a cooperative spectrum sensing network, we can choose the appropriate SUs as the sub-sampling channel by using a quantized distance. That is to say, the selected user must be within this quantized distance.

As can be seen from Figure 13, when $L\left(t_{d}\right)\geq 0.8$ and SNR≥15 dB, we can obtain a high recovery success rate of above 93%, and we only need no less than 20 collaborative users. This number is significantly less than the number of SUs required in [30]. In addition, when SNR= 20dB and m≥20, it can also achieve a high sensing accuracy under high transmission loss conditions. However, when SNR≤5 dB, the performance of the PSS-SaDMWC scheme will get worse. Meanwhile, when the loss gain becomes very small, the recovery success rate will also decrease significantly due to the destruction of the joint sparse property.

(5) Discussion of time complexity: The software environment for the experiment is: 64-bit Win7 operation system and Matlab 2017b. The hardware environment is an Intel(R) Core(TM) i5-4590 CPU @ 3.3 GHz and 8 GB RAM, and the PC used in the experiments was manufactured by Lenovo Inc. of Beijing, China. From the analysis of Cao’s research [49], we know that the magnitude of the time complexity of the whole sensing process is related to the sparsity order *K*, and *K* is directly related to the number of sub-bands *N*. Obviously, the number of reconfigurable sub-bands *N* is an important indicator of the reconstructed ability of the spectrum sensing algorithm. Therefore, we give the time complexity results of the simulation experiments for different *N*. 

The time overhead of MWC-based wideband spectrum sensing is mainly composed of two phases: (1) the time taken by the sub-Nyquist sampling process of the parallel channel; and (2) the time taken for the spectral support to be reconstructed. As shown in Table 6, the main time cost of wideband spectrum sensing is spent during the sub-Nyquist sampling phase, and the time spent on the support reconstruction phase is relatively brief.

Subsequently, it is worth noting that two types of sensing methods are proposed in the IEEE 802.22 standard for the type of the primary service: fast sensing and fine sensing [50]. For the public safety spectrum, the sensing period is required to be very small. However, for the TV band space where the spectrum usage varies over a much larger timescale, the real time requirements for the sensing period are not so strict [51]. Therefore, the results, as shown in Table 7, show that as long as we choose the appropriate cascading progressive levels and the appropriate algorithms, the time complexity of the proposed scheme is fully acceptable for both types.

## 5. Conclusions

In this paper, by exploiting the intrinsic properties of the sparse multi-bands signal and cascaded progressive operation-based support selection strategy, a self-adaptive distributed MWC wideband spectrum sensing scheme (PSS-SaDMWC) was proposed. The PSS-SaDMWC scheme can achieve self-adaptive blind wideband spectrum sensing in CR networks, and can significantly improve the recovery success rate of support and the recovery ability of time-varying support when there are fewer cooperative SUs. Moreover, the PSS-SaDMWC scheme took full use of the advantages of the MWC architecture to achieve centralized cooperative spectrum sensing without increasing hardware complexity. Theoretical analysis and numerical results showed that the proposed scheme can find a good balance between transmission interference and sensing accuracy. In addition, the reconstruction performance was improved by up to 34% in comparison with the best single selection scheme, and the sampling rate can be reduced to 16.9%, 11.8%, 9.2% and 8.2% of the Nyquist sampling rate when the SNR is equal to 5 dB, 10 dB, 15 dB, and 20 dB, respectively. 

## Figures and Tables

**Table 1:** List of the notations.

Notation	Meaning
Λ	Actual spectral support of the signal.
$\dot{\Lambda }$	Temporary support obtained in Algorithm 2.
$\hat{\Lambda }$	Estimated spectral support.
|Λ|	Potential of spectral support.
N	Number of sub-bands in the multi-band signal.
$B_{i}$	Bandwidth of the ith sub-band.
$f_{nyq}$	Nyquist rate of x(t).
$p_{i}(t)$	Periodic mixing signal.
Y	Sub-Nyquist sampling signal with the MWC.
K	Sparsity order of the signal.
$\hat{K}$	Estimated sparsity order.
${\left\|{\tilde{Z}}^{i\to }\right\|}_{2}$	Norm of each row vector for $\tilde{Z}$.
Φ	m×L measurement matrix or observation matrix.
$\Phi_{↓\hat{\Lambda }}$	Extracting column vectors from Φ according to $\hat{\Lambda }$.
ε	Decision threshold.
$E_{i}$	Energy coefficient of the ith sub-band.
$f_{i}$	Carrier frequency.
$\tau_{i}$	Time offset of the ith sub-band.
L	Spectrum slice number.
$f_{p}$	Spectral slice width, $f_{p}=f_{nyq}/L$.
$f_{s}$	Sampling rate at each channel, $f_{s}=qf_{p}$, with odd q.

**Table 2:** The comparison of the extraction and reconstruction ability.

Parameters	$\mathrm{SNR}\;=\;15\;\mathrm{dB},\;B\;=\;50\;\mathrm{MHz},\;f_{NYQ}=10\mathrm{GHz},\;m\;=\;15,\;$ N = 6, and L(td) = 0.9	
**Scheme**	RPMB	S-MUSIC
**Extraction ability**	45.4%	61.4%
**Recovery success rate**	7.6%	34%

**Table 3:** Simulation parameters.

Symbols	Value	Meanings
N	6	Number of sub-bands with energy (three pairs of bands)
$E_{i}$	{1,2,3}	Energy of the *i-*th sub-band
$B_{i}$	{50,50,50} MHz	Maximal width of each sub-band
$\tau_{i}$	{0.4,0.7,0.2}	Time offset of the *i-*th sub-band
$f_{nyq}$	10 GHz	Nyquist rate
L	195	Aliasing rate, or the spectrum slice number
M	195	Number of intervals in each period of $p_{i}\left(t\right)$
$f_{p}$	51.28 MHz	Spectral slice width, $f_{p}=f_{nyq}/L$
$f_{s}$	51.28 MHz	Sampling rate at each channel, $f_{s}=qf_{p}$, with odd q
$L_{j}(t_{d})$	0.9	Transmission loss gain

**Table 4:** Comparison of the maximum promoting rates.

Comparison	m = 19 SNR = 5 dB	m = 18 SNR = 10 dB	m = 15 SNR = 15 dB	m = 15 SNR = 20 dB
PSS-SaDMWC scheme vs. the best single selection scheme	23%↑	34%↑	22%↑	7.5%↑

**Table 5:** The comparison on the minimum number of SUs and minimum sampling rate needed.

Schemes	SNR = 5 dB		SNR = 10 dB		SNR = 15 dB		SNR = 20 dB	
	$m_{\min}$	$f_{\sum \min}/\mathrm{MHz}$	$m_{\min}$	$f_{\sum \min}/\mathrm{MHz}$	$m_{\min}$	$f_{\sum \min}/\mathrm{MHz}$	$m_{\min}$	$f_{\sum \min}/\mathrm{MHz}$
PSS-SaDMWC	33	1692.24	23	1179.44	18	923.04	16	820.48
The best single selection scheme	39	1999.92	27	1384.56	23	1179.44	18	923.04

**Table 6:** Average time spent during sub-Nyquist sampling and reconstruction with different *N*.

Number of Bands with Energy (*N*)	Parameters: m = 20, SNR = 15 dB, L(td) = 0.9, Repeat 500 Times, Progressive Levels = 2.						
	Average Time Spent on Sub-Nyquist Sampling Process and the Support Reconstruction (/s)						
	Sub-Nyquist Sampling	SOMP_DMWC	SwSOMP_DMWC	ReMBo_DMWC	RPMB_DMWC	S-MUSIC_DMWC	PSS-SaDMWC
2	0.1175	0.0009	0.0010	0.0030	0.0138	0.0003	0.0046
4	0.1196	0.0014	0.0020	0.0115	0.0566	0.0004	0.0051
6	0.1187	0.0026	0.0034	0.0203	0.1260	0.0007	0.0062
8	0.1180	0.0034	0.0033	0.0266	0.2162	0.0007	0.0057
10	0.1193	0.0048	0.0036	0.0064	0.1175	0.0007	0.0062

**Table 7:** The total time spent on different schemes with different *N*.

Number of Bands with Energy (*N*)	Parameters: m = 20, SNR = 15 dB, L(td) = 0.9, Repeat 500 times, Progressive levels = 2.					
	The Total Time Spent on Different Schemes (/s)					
	SOMP_DMWC	SwSOMP_DMWC	ReMBo_DMWC	RPMB_DMWC	S-MUSIC_DMWC	PSS-SaDMWC
2	0.1184	0.1185	0.1205	0.1313	0.1178	0.1221
4	0.1210	0.1216	0.1311	0.1762	0.1200	0.1247
6	0.1213	0.1221	0.1390	0.2447	0.1194	0.1249
8	0.1214	0.1213	0.1446	0.3342	0.1187	0.1237
10	0.1241	0.1229	0.1257	0.2368	0.1200	0.1255

**Figure A1:**
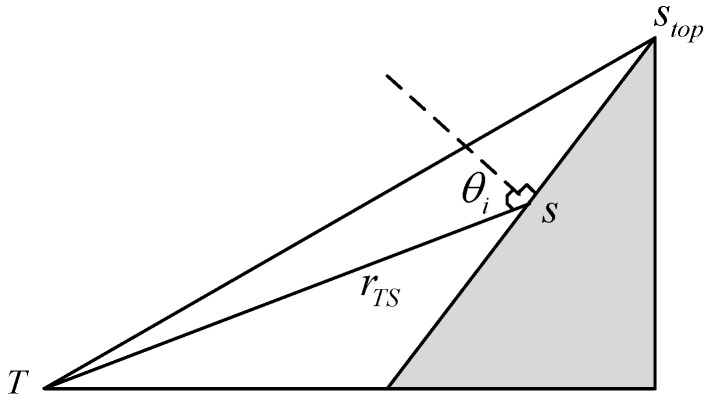
Schematic diagram of the scatterer and the left view of the scatterer slope.

## Appendix A

Considering the diffuse reflection effect, a general formula for radio wave propagation loss is given in [40], which can be expressed as:

(A1) $$L(t_{d})=\frac{P_{R}(t=(r_{TS}+r_{SR})/c)}{P_{T}(t=0)}=\frac{\lambda^{2}\sigma }{{\left(4\pi \right)}^{3}r_{TS}{}^{2}r_{SR}{}^{2}}$$

where σ denotes the effective diffuse reflection area. Assuming that the slope of each point on the target scatterer is the same, σ can be expressed as:

(A2) $$\sigma =\sigma^{0}A$$

According to Lambert radiator model theory [52], we have:

(A3) $$\sigma^{0}=\gamma \cos\theta_{i}$$

where γ is the reflectivity of scatterers, $\theta_{i}$ represents the angle between ***TS*** and the diffuse surface normal, and ***TS*** represents the vector between radio frequency (RF) transmitter **T** and any scattering point **S**. Figure A1 shows the schematic diagram. If the slope of each point on the target scatterer is not the same, the effective diffuse reflection region can be approximately represented as:

(A4) $$\sigma =\sigma^{0}dA$$

Combining (A1) and (A4), we can get Equation (7).
